# Eosinophilic Enterocolitis Diagnosed by Means of Upper Endoscopy and Colonoscopy with Random Biopsies Treated with Budenoside: A Case Report and Review of the Literature

**DOI:** 10.5402/2011/608901

**Published:** 2011-01-17

**Authors:** Ghulamullah Shahzad, Duane Moise, Seth Lipka, Kaleem Rizvon, Paul J. Mustacchia

**Affiliations:** Nassau University Medical Center, East Meadow, NY 11554, USA

## Abstract

Intense infiltration of gastrointestinal and colonic mucosa with eosinophils or acidophilic gastroenteritis (EG) is a relatively uncommon picture for a pathologist endoscopist especially outside the pediatric age group and is highly suggestive of an ongoing chronic inflammatory process. Existing literature projected a hypothetical association with allergy but the exact pathophysiology is still unknown. Association with malabsorption, protein losing enteropathy, and refractory ulcers with gastrointestinal bleeding makes the clinical presentation more complicated. We present a unique case of diarrhea and abdominal pain in the clinical presentation with associated peripheral eosinophilia, asthma, and gastroesophageal reflux disease (GERD). The patient's symptoms abated after initiation of budesonide.

## 1. Case Report

A 45-year-old white female reported to the emergency department with chief complaints of abdominal pain and shortness of breath. These symptoms were associated with a 1-week history of nausea, vomiting, abdominal pain, and diarrhea. The patient described the diarrhea as watery, with a stool frequency of 10–20 movements daily, without blood. The vomiting typically occurred within 15 minutes of eating and was greenish with no evidence of blood. The symptoms occurred the night after construction began at her work place; she was a clerical postal service employee. The construction appeared to trigger an asthma exacerbation. She denied recent travel, sick contacts, consuming raw seafood, fever/chills, or eating out. She denied any allergies to medicines or foodstuff. She had a past history of GERD and asthma. She denied alcohol or recreational drug use but she smoked 1-2 cigarettes a day for the past 6 years. Current medications included Pantoperazole and Albuterol inhaler.

On physical examination, the patient was afebrile and vitals were within normal limits. The patient had dry mucous membranes. Abdominal exam revealed diffuse mild tenderness. Complete blood count showed a white cell count of 11.6 (nl 4.0–10.0). Complete metabolic panel was within normal limits. Fecal occult blood was negative. Stool culture for ova and parasites, C.difficile toxin, leukocytes, *Salmonella*, *Shigella*, *Campylobacter*, Yesinia, and E. Coli 0157:H7 were all negative. IgE mold allergens were also negative. Serum IgE was within normal range. Peripheral eosinophils were elevated with an absolute eosinophil count peaking at 4.8 during hospital stay (NL 0–0.5 k/mm^3^), and computed tomography (CT) of the abdomen and pelvis revealed no abnormalities or inflammation. 

**Figure 1:**
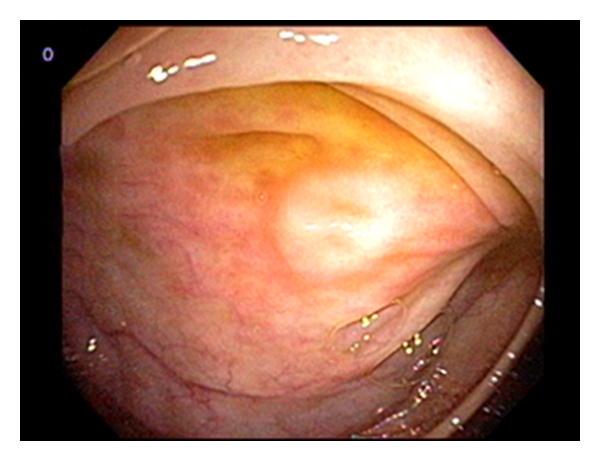
Unremarkable cecal mucosa.

**Figure 2:**
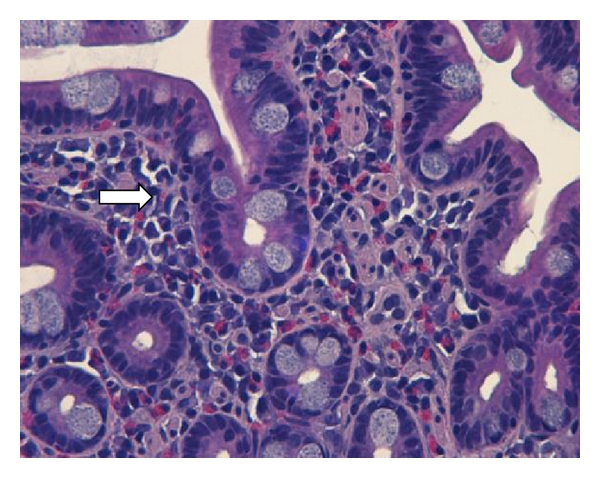
Eosinophilic infiltration.

The patient was admitted under the working diagnosis of gastroenteritis and treated with intravenous fluids and antibiotics. There was no improvement over the next 3-day duration. Upper endoscopy and colonoscopy showed signs of inflammation in the gastric antrum with no other macroscopic evidence of inflammation in the upper and lower gastrointestinal tract (Figure 1). Antral biopsy was unremarkable and negative for *H. Pylori*. Small bowel biopsy showed signs of inflammation with high level of eosinophilic infiltrates (>15/hpf) (Figure 2). Even though the colon appeared macroscopically unremarkable, random mucosal biopsies showed microscopic pathology in the right and left colon. There were greater than 15 eosinophils per high-power field in the colonic mucosal biopsy specimens. Rectal biopsies were unremarkable. A diagnosis of eosinophilic enterocolitis was made, and the patient was discharged with conservative management with an outpatient clinic visit within a week of discharge. Since the patient's symptoms persisted, a trial of budesonide was initiated which resulted in complete resolution of her symptoms.

## 2. Discussion

Eosinophilic enterocolitis represents a subset of a broader disease group that includes eosinophilic esophagitis, gastritis, enteritis, and colitis, commonly referred to as the eosinophilic gastrointestinal disorders [1].

Eosinophilic gastroenterocolitis is an extremely rare disease, with less than 200 patients described; the largest published series includes only 40 patients [2, 3]. The first reported case was originally described by Kaijser in 1937, as a rare spectrum of gastrointestinal disorders characterized by inflammation rich in eosinophils without evidence of a known cause for eosinophilia [4]. Currently, the mean duration between symptom onset and diagnosis in patients with eosinophilic gastroenteritis is 4.1 ± 5.7 years [5]. The disease can affect patients of any age, but is more commonly seen in the third through fifth decades with a male predominance [2, 6]. Eosinophilic gastroenteritis should be considered when a patient presents with unexplained gastrointestinal symptoms that cannot be defined by parasitic or other gastrointestinal diseases. Diagnosis is defined by three criteria: (a) the presence of GI symptoms, (b) biopsies showing eosinophilic infiltration of one or more areas of the GI tract (or with characteristic radiological findings with peripheral eosinophilia), and (c) no evidence of parasitic or extraintestinal disease [2]. 

A peripheral eosinophilia may be helpful but is not necessarily a universal finding, and it may be absent in 20%–90% of cases [7, 8]. Klein subdivided the disease based on the extent of bowel wall most infiltrated by eosinophils: mucosa, muscularis, and subserosa [9]. Due to the patchy infiltrate of the disease throughout the GI tract, eosinophilic gastroenteritis cannot be ruled out even if all biopsies are negative. In one study, 5 out of 40 endoscopic biopsies, missed the eosinophilic infiltrate [2]. A study using CT to look for areas of inflammation, as well as surgical full thickness biopsies may help localize areas for biopsies [10]. The most common radiographic finding seen in eosinophilic gastroenteritis is diffuse nodular and irregular fold thickening in the distal stomach and in the proximal small bowel [11, 12]. 

The cause of eosinophilic gastroenteritis remains unknown. Seventy-five percent of affected patients have a history of allergy or atopy [5]. A case report demonstrated accumulation of mast cells in the interstium of the colon wall after immunohistochemical staining for the mast cell enzyme tryptase suggesting the pathogenic role of IgE [13]. Another study showed possible pathogenesis involving the eosinophilic chemoattractants inerleukin-5 and eotaxin [14]. Therapy of eosinophilic gastroenteritis is limited to case reports and retrospective studies; therefore, without prospective and randomized therapeutic trials, treatment remains purely empirical and based on clinical manifestations. An empiric elimination diet, with the assistance of a dietitian trained in eosinophilic GI disorders, may provide successful treatment [15]. Chehade et al. reported that children who were placed on an elemental diet showed a significant decrease in symptoms [16]. In more severe cases, oral Prednisone (20–40 mg/day), with a rapid taper, may provide success. In one study involving 13 patients all had reported successful resolution of symptoms within 2 weeks [15]. Other therapies have been tried including sodium chromoglycate, ketotifen [17], montelukast [18], and Th2 cytokine inhibitor suplast tosilate [19]. However, their long-term effectiveness is questionable and requires more prospective clinical trials. Foroughi and colleagues reported success in an open-label study of nine patients treated with omalizumab, a monoclonal anti-IgE antibody [6]. Patients with relapsing and refractory disease are often placed on low-dose, long-term steroids, or immunosuppressive therapy. Few studies have followed patients with eosinophilic gastroenteritis for long term, but long-term treatment is often ineffective.

In summary, Eosinophilic enterocolitis may represent an underappreciated condition. The recognition of eosinophilic enterocolitis constitutes a challenge to hospitalists, gastroenterologists, and the physician community at large.
